# Immune Checkpoint Markers in Neuroendocrine Carcinoma of the Digestive System

**DOI:** 10.3389/fonc.2020.00132

**Published:** 2020-02-28

**Authors:** Jiazhang Xing, Hongyan Ying, Ji Li, Yang Gao, Zhao Sun, Jiarui Li, Chunmei Bai, Yuejuan Cheng, Huanwen Wu

**Affiliations:** ^1^Department of Medical Oncology, Peking Union Medical College Hospital, Chinese Academy of Medical Sciences, Beijing, China; ^2^Department of Pathology, Peking Union Medical College Hospital, Chinese Academy of Medical Sciences, Beijing, China

**Keywords:** immune cell infiltration, microsatellite instability, neuroendocrine carcinoma, programmed cell death ligand 1, tumor mutational burden

## Abstract

Digestive system neuroendocrine carcinomas (NECs) are rare neoplasms originating from neuroendocrine cells with a poor prognosis and limited effective treatments. Programmed cell death protein 1/ligand 1 (PD-1/PD-L1) blockade has been used in the management of more than 10 solid tumors and has achieved promising clinical outcomes. PD-L1 expression, immune cell infiltration, tumor mutational burden (TMB), and microsatellite instability (MSI) are all verified biomarkers that can predict the response to anti-PD-1/PD-L1 therapy. Here, we investigated PD-L1 expression and immune cell infiltration density by immunohistochemical (IHC) staining of tumor samples from 33 patients with digestive system NECs. Tumor and paratumor normal samples from 31 of these patients underwent whole-exome sequencing to evaluate TMB and the MSI-high (MSI-H) status. In total, 29.0% of digestive system NECs had positive PD-L1 expression according to the tumor proportion score (TPS). Infiltration of CD3^+^, CD8^+^, and CD68^+^ cells was observed in 69.7, 27.3, and 54.5% of patients, respectively. The TMB value for patients sequenced ranged from 0.57 to 11.75 mutations/Mb, with a median of 5.68 mutations/Mb. mSINGS, MSIsensor, and MSIseq were used to analyze the MSI status according to the sequencing data, and in our evaluation, no MSI-H status was detected. Our data might indicate a limited potential of anti-PD-1/PD-L1 monotherapy in digestive system NECs, although clinical trials are warranted.

## Introduction

Neuroendocrine neoplasms (NENs) are defined as neoplasms composed of cells with a neuroendocrine phenotype or features. Pathologically, NENs are graded as grade 1, 2, and 3 according to the 2010 WHO classification. Poorly differentiated grade 3 NENs are referred to as neuroendocrine carcinomas (NECs) (1). Digestive system NECs are the most common extrapulmonary NECs, with an incidence rate of 0.4 per 100,000 (2). The prognosis of digestive system NECs is very poor; 60.8% of patients have distant metastases at diagnosis, and the 5-year survival rate is 13.1% (2). Due to their rarity, treatment for digestive system NECs is extrapolated from research data of small cell lung cancer (SCLC). However, significant clinical and pathological differences between SCLCs and digestive system NECs have been discovered. At present, first-line chemotherapy for digestive system NECs including cisplatin or carboplatin with etoposide or irinotecan has a controversial response rate of 40–60%, and no second-line regimen has been established (3).

Programmed cell death ligand 1 (PD-L1), an immune inhibitory protein, is often upregulated in tumor cells by interferon-γ (IFN-γ) secreted from effector T cells when the tumor antigen was recognized. By interacting with programmed cell death protein 1 (PD-1), PD-L1 can suppress many immune cell functions, especially T cell activation. Then, PD-1 blockade is believed to normalize antitumor immunity (4). PD-1/PD-L1 monoantibodies, such as nivolumab, pembrolizumab, and atezolizumab, have already been approved by the Food and Drug Administration (FDA) for the treatment of melanoma, non-small cell lung cancer (NSCLC), head and neck squamous cell carcinoma (HNSCC), urothelial cancer, and more than 10 other cancers.

The determinants for the clinical benefits of PD-1/PD-L1 blockade have been demonstrated in several tumors. Positive immunohistochemical (IHC) expression of PD-L1 proved to be a predictive biomarker of efficacy in several solid tumors, including NSCLC, urothelial cancer (5), and HNSCC (6). Tumor-infiltrating lymphocytes (TILs) are another important factor in PD-1 blockade. In melanoma, high pretreatment TILs are associated with a good response to immunotherapy and survival, and the CD8 cell count has a significant predictive value (7, 8). Tumor mutational burden (TMB) and microsatellite instability (MSI) have also been verified as biomarkers in the prediction of anti-PD1/PD-L1 monotherapy outcomes (9–15). An MSI-high (MSI-H) status or a high TMB indicates many somatic mutations, which could be recognized by the immune system (16). A significant correlation between TMB and the PD1 inhibition response rate, with a coefficient of 0.74, was demonstrated in previous research (17). In 2017, the FDA approved pembrolizumab for the conditional treatment of unresectable or metastatic, MSI-H, or mismatch repair-deficient solid tumors (18).

Due to the rarity of NECs, clinical trials of PD-1/PD-L1 blockade are still in progress, and few studies have thoroughly evaluated immunotherapy potential in digestive system NECs. In this study, we investigated PD-L1 expression, immune cell infiltration density, the MSI-H status, and TMB in 33 patients with digestive system NECs to demonstrate the immune microenvironment of digestive system NECs and help predict the effectiveness of PD-1/PD-L1 blockade in NECs.

## Materials and Methods

### Study Design

Sixty-four formalin-fixed paraffin-embedded tissue samples (including tumor and paratumor tissue) from 33 patients pathologically diagnosed with an NEC according to WHO 2010 criteria between 2009 and 2019 at Peking Union Medical College Hospital (PUMCH) were included in our study. Samples were obtained from the tumor bank of PUMCH. This study was carried out in accordance with the recommendations of the ethics committee of PUMCH with written informed consent from all subjects. The samples of 32 patients were from the primary sites, and one was from a metastatic site. All of the samples were obtained by surgery. Two expert pathologists reviewed all the patients to ensure inclusion. IHC staining was used to evaluate the expression of PD-L1, CD3, CD8, and CD68. Whole-exome sequencing was employed to sequence each pair of tumor and paratumor normal tissue from 31 patients. Histopathologic characteristics, including the tumor site, pathological description, lymph node status, and IHC markers (such as synaptophysin, chromogranin A, and the Ki-67 index), were collected from the original pathology reports. Every patient had intact medical history records at PUMCH. Clinical characteristics, including sex, age at diagnosis, systemic management, and follow-up records, were obtained. All the patients were restaged pathologically by surgery and post-surgery pathology records according to the 8th AJCC TNM classification criteria.

### Immunohistochemical Analysis

**Figure 1:**
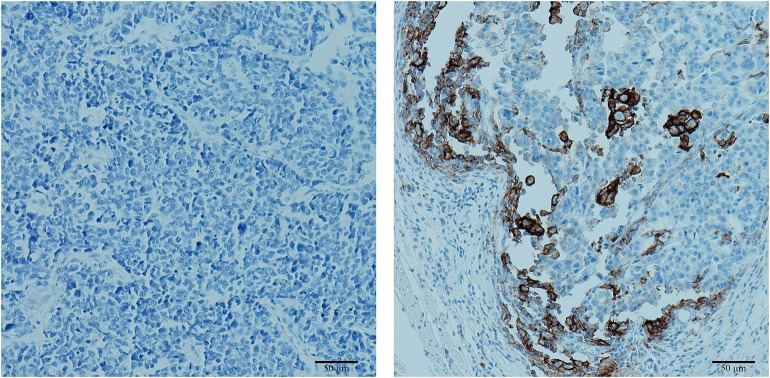
Representative images of programmed cell death ligand 1 (PD-L1) immunohistochemical (IHC) staining. Left: negative PD-L1 expression with a tumor proportion score of 0%. Right: positive PD-L1 expression with a tumor proportion score of 15%.

Four-micrometer-thick sequential tumor sections were obtained from paraffin-embedded tissue samples and used for IHC analysis. An automated staining system (Dako Autostainer Link 48, PUMCH) was used for PD-L1 staining. CD3, CD8, and CD68 were stained manually by experienced technicians. The antibodies used in IHC staining were antibodies against PD-L1 (clone 22C3, dilution 1:50; M365329-8CN, Dako), CD3 (T cell lymphocytes; dilution 1:100; GB13014, Servicebio), CD8 (cytotoxic T cells; dilution 1:500; GB11068-1, Servicebio), and CD68 (macrophages; dilution 1:200; GB14043, Servicebio). IHC staining results were evaluated under light microscopy by two experienced pathologists who were blinded to all original data. In the PD-L1 staining evaluation, the tumor proportion score (TPS) system was used. Tumor cells with partial or complete cell membrane PD-L1 staining were scored regardless of the staining extent. Cytoplasmic staining and staining on immune cells or necrotic cells were disregarded. The proportion of PD-L1-positive cells in the whole tumor area was recorded for each sample, and positive expression was recorded when the staining proportion was >1%. Semiquantitative scores were used to evaluate the staining density of CD3, CD8, and CD68 as 0 (no positive cells), 1 (staining cell infiltration <25% of the stromal area), 2 (staining cell infiltration 25–49% of the stromal area), or 3 (staining cell infiltration >50% of the stromal area). Low infiltration (score 0–1) and high infiltration (score 2–3) samples were subjected to statistical analysis (19). Representative PD-L1 stains of the investigated tissues are shown in Figure 1.

### Tumor Mutational Burden and Microsatellite Instability Assessment

Tumor and paratumor normal DNA from 31 patients was obtained from paraffin-embedded tissues using five representative 10-mm-thick sections of tumor samples. Whole-exome sequencing was performed based on alignment to the reference human genome GRCh37, variants and somatic mutations were detected using the Genome Analysis Tool Kit (GATK, version 4.0.6.0, The Broad Institute, Cambridge, MA). Likely alterations or known oncogenic drivers and germline polymorphisms were excluded. TMB was calculated by dividing the total number of somatic mutations by the length of the capture region. mSINGS (20), MSIsensor (21), and MSIseq (22) were used to analyze the MSI status according to the sequencing data. The MSI-H status was defined as when more than two software programs showed MSI-H.

### Statistical Methods

Fisher's exact test, the Mann–Whitney test and the Kruskal–Wallis test were used to detect differences in categorical and continuous variables between groups of patients. A Kaplan–Meier curve was applied to the survival analysis. The statistical software program SPSS version 24 was used for all analyses.

## Results

### Patient Characteristics

Thirty-three patients were included in our study. Their basic characteristics are listed in Table 1. Twenty-one males and 12 females were enrolled, and their median age at diagnosis was 65 years (range from 20 to 81 years). The stomach was the most common primary site in these patients (21 patients, 63.6%), and six patients' primary sites were the pancreas (18.2%). Other primary sites included the esophagus, small intestine, gallbladder, and colon. Most patients were in stage II or III pathologically, with percentages of 36.4 and 45.5%, respectively. Four patients presented with synchronous distant metastasis at diagnosis, and 12 patients developed metachronous distant metastasis after surgery. In the Kaplan–Meier survival analysis, patients with distant metastasis had significantly shorter overall survival (OS) than patients without distant metastasis (*p* = 0.017). Six patients had two or more metastatic sites. The liver was the most common metastatic site (75.0%), and other sites included the lung, peritoneum, pancreas, kidney, spleen, ovary, adrenal gland, and brain. All the patients were followed up after their surgery until June 2019; three patients were lost to follow-up, and 13 patients were still alive.

**Table 1:** Baseline characteristics of 33 patients with a digestive system neuroendocrine carcinoma.

	***n***	**%**
Gender		
Male	21	63.6
Female	12	36.4
Age, years		
Median, range	65 (20-81)	
Primary tumor site		
Esophagus	3	9.1
Stomach	21	63.6
Small Intestine	1	3.0
Colon	1	3.0
Gallbladder	1	3.0
Pancreas	6	18.2
TNM		
I	2	6.1
II	12	36.4
III	15	45.5
IV	4	12.1
Distant metastasis	16	48.5
Synchronous	4	12.1
Metachronous	12	36.4
Site of distant metastasis		
Liver	12	36.4
Kidney	4	12.1
Lung	2	6.0
Ovary	1	3.0
Peritoneum	1	3.0
Pancreas	1	3.0
Adrenal gland	1	3.0
Spleen	1	3.0
Brain	1	3.0

### Programmed Cell Death Ligand 1 Expression in Digestive System Neuroendocrine Carcinomas

**Figure 2:**
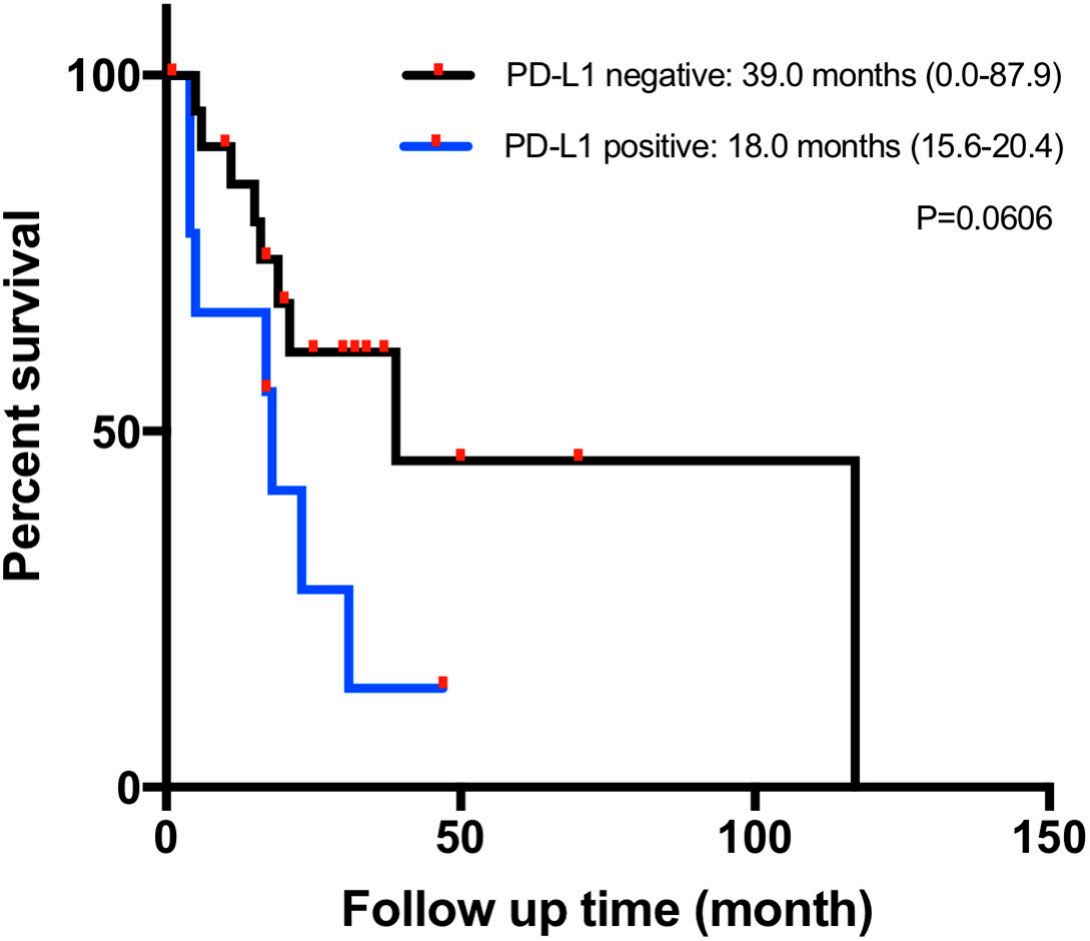
Kaplan–Meier overall survival (OS) curves in patients with gastrointestinal neuroendocrine carcinomas (NECs) based on the programmed cell death ligand 1 (PD-L1) immunohistochemical (IHC) staining status.

Of the 33 tumor samples, two were detached from the slides in PD-L1 staining and excluded from PD-L1 expression-related analyses. Nine of 31 patients (29.0%) had positive PD-L1 expression according to the TPS evaluation, although the TPS for all detected sample was under 15%. PD-L1 expression was not associated with sex, the primary site, the TNM stage, the Ki-67 index, or other investigated markers. Patients with PD-L1-positive tumors tended to have a shorter median OS of 18.0 months (95% CI 15.6–20.4) than those with PD-L1-negative tumors (39.0 months, 95% CI 0.0–87.9), but without reaching statistical significance (*p* = 0.061) (Figure 2). Information on both positive and negative PD-L1 expression patients is shown in Table 2.

**Table 2:** Characteristics of and immune markers in patients with positive and negative programmed cell death ligand 1 (PD-L1) expression.

**Category**	**PD-L1 positive**	**PD-L1 negative**	**Total**
Gender, *n* (%)			
Female	4 (44.4%)	7 (31.8%)	11 (35.5%)
Male	5 (55.6%)	15 (68.2%)	20 (64.5%)
Median age at diagnosis (range)	70 (46–80)	60.5 (20–81)	65 (20–81)
Primary sites, *n* (%)			
Stomach	8 (88.9%)	12 (54.5%)	20 (64.5%)
Pancreas	1 (11.1%)	5 (22.7%)	6 (19.4%)
Other sites	0 (0.0%)	5 (22.7%)	5 (16.1%)
Median Ki-67 index (range)	80% (50–95)	80% (30–90)	80% (30–95)
TNM stage, *n* (%)			
I	1 (11.1%)	1 (4.5%)	2 (6.5%)
II	3 (33.3%)	9 (40.9%)	12 (38.7%)
III	5 (55.6%)	9 (40.9%)	14 (45.2%)
IV	0 (0%)	3 (13.6%)	3 (9.7%)
CD3^+^ Cell infiltration, *n* (%)			
High infiltration	6 (66.7%)	3 (13.6%)	9 (29.0%)
Low infiltration	3 (33.3%)	19 (86.4%)	22 (71.0%)
CD8^+^ cell infiltration, *n* (%)			
Infiltration	4 (44.4%)	5 (22.7%)	9 (29.0%)
No infiltration	5 (55.6%)	17 (77.3%)	22 (71.0%)
CD68^+^ Cell infiltration, *n* (%)			
High infiltration	3 (33.3%)	3 (13.6%)	6 (19.4%)
Low infiltration	6 (66.7%)	19 (86.4%)	25 (80.6%)
Median tumor mutation burden (range)	6.58 (3.92–11.75)	5.18 (0.57–11.33)	5.915 (0.57–11.75)

### Immune Cell Infiltration

All 33 tumor samples were stained and evaluated for the immune markers CD3, CD8, and CD68. According to our semiquantitative scores, CD3^+^ T cell infiltration was observed in 23 patients (69.7%), with nine patients detected as high infiltration; CD8^+^ cytotoxic cell infiltration was observed in nine patients (27.3%), and all were detected as low infiltration; CD68^+^ macrophage cell infiltration was observed in 18 patients (54.5%), with six patients detected as high infiltration. CD3^+^ T cell infiltration was correlated with CD8^+^ T cell and CD68^+^ macrophage cells' infiltration, with p values of 0.095 and 0.005, respectively. No association of immune cell infiltration with patient characteristics was found according to Fisher's exact test. In the survival analysis, difference in OS was not observed between different infiltration levels of CD3^+^, CD8^+^, or CD68^+^ cells, separately.

### Tumor Mutational Burden and Microsatellite Instability-High Analysis

Thirty-one patients with both tumor and paratumor normal tissues were sequenced and evaluated for TMB and the MSI-H status. Two patients were not sequenced due to a lack of normal tissue. The TMB value ranged from 0.57 to 11.75 mutations/Mb, with a median of 5.68 mutations/Mb. There was no correlation between the TMB value and clinical characteristics or OS. The median TMB was 6.58 mutations/Mb for PD-L1-positive samples and 5.18 mutations/Mb for PD-L1-negative samples. In PD-L1-positive samples, TMB tended to be positively correlated with the TPS score, although the difference did not reach statistical significance (*p* = 0.058) in the Spearman test. In our evaluation, no MSI-H status was detected.

## Discussion

In our study, we investigated PD-L1 IHC expression, immune cell infiltration, the MSI-H status, and TMB in digestive system NECs.

Nine patients (29.0%) had positive PD-L1 expression. The positive PD-L1 expression percentages in digestive system NEC tumor cells were reported previously as 28.6% (6/21) (23), 14% (5/37) (24), 37.5% (6/16) (25), 100% (9/9) (26), and 41.17% (7/17) (27), although the primary site constitutions were different in these studies. Survival was numerically different between patients with PD-L1-positive and PD-L1negative tumors, but this finding did not reach statistical significance. In Kim's study of metastatic gastroenteropancreatic neuroendocrine tumors (GEP-NETs), which included 15 NETs and 17 NECs, the impact of PD-L1 expression on OS was demonstrated with a median OS of 16.0 and 24.8 months in patients with PD-L1-positive and PD-L1-negative NETs, respectively (*p* = 0.037) (27). In another study on gastric NECs, patients with PD-L1 expression tended to have a shorter OS time than patients lacking PD-L1 expression (*p* = 0.016), and PD-L1 expression was identified as an independent prognostic factor (28).

Some factors can affect PD-L1 IHC staining and lead to misleading results. Based on our experience, in this study, we preliminarily used two different antibodies (clone SP142, Roche, and clone 28-8, ab205921, Abcam) but failed to obtain the result of the expression. A similar situation was also reported in another study on digestive system NETs (29). Although it was reported that different antibodies can lead to a concordant result in NSCLC (30), it is still questionable whether the antibody can be used in other tumors, especially in rare tumors, such as digestive system NECs, with few studies. Meanwhile, the variability of the staining method (manual or platform) and the lack of a standard staining procedure in NECs also contributed to the inaccuracy in PD-L1 testing. The heterogeneity of PD-L1 expression may also influence the IHC results (e.g., differences between biopsy and whole resection specimens) (31), and the potential influence of therapy should be noted (32).

We observed T cell and macrophage infiltration in more than half of the tumor samples. Approximately one third of patients showed cytotoxic T cell infiltration but all with low infiltration level. Several studies had explored lymphocyte infiltration in NECs or NETs, although the criteria for evaluation were varied, and the results were not totally consistent (23, 28, 29, 33). In our study, patients with a low T cell or cytotoxic T cell infiltration in tumors did not show any significant difference in survival compared with patients with a high infiltration. Few studies have reported the relationship between the tumor infiltration of immune cells and OS in digestive system NECs. One study reported that a low CD8^+^ cell infiltration might indicate poor OS in gastric NECs (*p* = 0.065) (28). In another study focusing on pancreatic NETs, reduced disease-free survival was proved to be significantly related to low intratumor CD8^+^ T cells (33).

In our research, most patients had low or intermediate TMB (<20 mutations/Mb) (34), with a median of 5.68 mutations/Mb. Before our study, the research of Chalmers et al. (35) showed that the median TMB was 2.7 and 3.7 mutations/Mb for pancreatic and colon NEC, respectively, although the definition of NEC was not clearly illustrated.

None of the patients had an MSI-H status in our research. MSI was detected in ~13.2% of patients with a GEP-NEC (7/53) in the study of Sahnane et al. (36), and in a meta-analysis of GEP-NECs, MSI-H was reported in ~10% of gastric and colorectal NECs (37). This inconsistency might mainly be attributed to the MSI-H status tested in our study. Studies have indicated that under conventional methods, the MSI-low (MSI-L) status is not distinguished among the MSI status, and between MSI-L and microsatellite stable cancers, there are no significant differences in the overall mutational burden (38). Therefore, it was more reasonable to evaluate MSI-H, but in some previous studies, the MSI-H status was not classified clearly. Additionally, the colorectal origin has accounted for a large portion of the primary sites in early studies, while our study mainly included NECs originating from the stomach and pancreas, which can also lead to inconsistency.

All the markers investigated in the current study were revealed as predictive biomarkers for the anti-PD-1/PD-L1 monotherapy response. From the perspective of PD-L1 expression and TILs, ~19.4% (6/31) of patients in our study have type I cancers with a positive PD-L1 expression and a high T cell infiltration (PD-L1+TILs+) and potentially benefit from anti-PD-1/PD-L1 monotherapy according to the TILs/PD-L1 status classification of Teng et al. (39). Meanwhile, all the samples in our study showed low or medium TMB status and MSI-L status. These data might indicate that the effectiveness of anti-PD-1/PD-L1 monotherapy in digestive system NECs is limited, which was in accordance with some preliminary clinical data (23). To date, no large-scale clinical trial results of anti-PD1 therapy in NECs are available, although some trials are ongoing (NCT03901378, NCT03136055, NCT03147404, NCT03591731, and NCT03728361).

There are also some limitations to our study. First, due to the rarity of NECs, our study was based on a limited sample size. For digestive system NECs, a low incidence rate also brings difficulty in the diagnosis. Second, none of the patients in our study had ever been treated with anti-PD-1/PD-L1 therapy; thus, the correlations of the markers we studied with the efficacy of anti-PD-1/PD-L1 therapy remain to be explored in the future.

In conclusion, our study contributes to the understanding of the immune microenvironment and mutational landscape of digestive system NECs. This understanding should help to improve predictions of the impact of PD-1/PD-L1 blockade in patients with digestive system NECs.

## Data Availability Statement

The data that support the findings of this study have been deposited in the CNSA (https://db.cngb.org/cnsa/) of CNGBdb with Accession No. CNP0000885.

## Ethics Statement

The studies involving human participants were reviewed and approved by the ethics committee of Peking union medical college hospital. The patients/participants provided their written informed consent to participate in this study.

## Author Contributions

YC, HW, HY, and JX contributed to the conception and design of the study. ZS and CB were involved in planning and supervising the work. JX, JiL, YG, and JiaL performed the experiments and analyzed the data. HY and JX wrote the first draft of the manuscript. All authors contributed to manuscript revision and read and approved the submitted version.

### Conflict of Interest

The authors declare that the research was conducted in the absence of any commercial or financial relationships that could be construed as a potential conflict of interest.
